# Cytological Aspects on the Effects of a Nasal Spray Consisting of Standardized Extract of Citrus Lemon and Essential Oils in Allergic Rhinopathy

**DOI:** 10.5402/2012/404606

**Published:** 2012-12-09

**Authors:** Lydia Ferrara, Daniele Naviglio, Arturo Armone Caruso

**Affiliations:** ^1^Department of Pharmaceutical and Toxicological Chemistry, Faculty of Pharmacy, University of Naples Federico II, Via Domenico Montesano 49, 80131 Naples, Italy; ^2^Department of Food Science, Faculty of Agriculture, University of Naples Federico II, Via Università 100, 80055 Portici (Naples), Italy; ^3^ENT Department, AIAS Structure of Afragola, Contrada Leutrec snc, 80021 Naples, Italy

## Abstract

In this paper, a new formulation of nasal spray was set up based on the extract of lemon pulp, obtained by using a new solid-liquid technology of extraction, added to pure *Aloe* juice, soluble propoli, and essential oils of *Ravensara* and Niaouly. It was tested in a clinical study in which 100 subjects were recruited for a period of one month. Nasal scraping was used for collecting samples and after the application of the May-Grünwald Giemsa standard technique, glass slides were analysed by using optical microscope with a 1000x oil immersion. A control group constituted of ten people was recruited as control and this group was administered with physiological solution (saline solution). The comparison of results obtained before and after the application of nasal spray showed a total reduction of eosinophils granulocytes and mast cells; clinical data were confirmed by improvement of clinical pictures of patients. The lemon-based nasal spray was a good alternative to conventional medicine for the treatment of perennial and seasonal allergic and vasomotor rhinopathy.

## 1. Nasal Cytology

Nasal cytology is of remarkable importance in the study of rhinosinus diseases, especially the vasomotor rhinitis (VMR), as it represents valuable means of differential diagnosis between allergic/nonallergic diseases and bacterial/viral infections. It is a popular and proven method, considering that it dates back to 1889, when Gollash [1] identified the numerous eosinophils in the nasal secretion from an asthmatic patient and attributed their presence a key role in the pathogenesis of asthma. The nasal cytodiagnosis was actually encouraged by the study of Eyermann [2] in 1927, who identified the eosinophils in the nasal exudate of allergic patients and underlined its diagnostic importance. Since then, lots of researchers have focused their attention on cytology and particularly on the presence of different types of inflammatory cells in nasal diseases [3, 4]. Different factors have contributed to the increased interest in cytological study of the nasal mucosa, making this procedure more widespread: the sampling is easy to perform and minimally invasive, allowing the examination to be repeated, as often required in the follow-up visits in the case of vasomotor disorders and for monitoring the effectiveness of some treatments. Some studies have proved that the rhinocytogram of patients with allergies varies according to the topical nasal steroid treatment. Meltzer et al. [5] and Small [6] have shown that fluticasone dipropionate and beclomethasone dipropionate are able to effectively control the symptoms of perennial and seasonal allergic and vasomotor rhinopathy and to induce cytological changes with a significant reduction in the number of eosinophils and basophils in the nasal mucosa. Cassano et al. showed that the anti-inflammatory effect of topical corticosteroid is doubtlessly proven by the reduction in the immune-inflammatory components observed on the rhinocytogram [7]. The cortisone therapy, despite being effective in most cases, presents disadvantages related to side effects after prolonged use; it is not tolerated by allergic individuals; it may not be used during pregnancy and lactation; finally, it may not be used by children under the age of 12.

The purpose of this study was to create a nasal spray based on lemon pulp extract, in light of the pharmacological properties of lemon, and to evaluate its therapeutic efficacy in different forms of rhinitis.

 In this study, the lemon extract has been prepared using an innovative solid-liquid extraction method, the Extractor Naviglio or Dynamic Fast Solid-Liquid Extractor [8]; this device features an innovative solid-liquid extraction technique that allows solid matrices, containing substances that can be extracted in organic or inorganic solvent and mixtures thereof, to be quickly extracted [9].

The experiment was conducted on 100 patients after completion of medicolegal procedures for a period of one month. The results have been reported in terms of subjective scores at the beginning and at the end of therapy and compared with the rhinocytological reports.

## 2. Methods

May-Grünwald-Giemsa Reagent (REAGENA Ltd., Toivala, Finland) and Nikon Eclipse 200 Nasal Scraping Microscope (Nikon Instruments S.p.a., Florence, Italy) were used in experimental trials; Lemon extract 1% titrated with citric acid 6%; Pure *Aloe* juice, and essential oil of *Ravensara*, soluble Propoli WSEP-70, Essential oil of Niaouly (Intermedia Synergie s.r.l., Cernobbio, Como, Italy) were used in the preparation of nasal spray.

 The extract obtained using the Extractor Naviglio had a pH between 3 and 3.5 and a citric acid content that ranges from 6% to 7% (w/w); the juice of *Aloe barbadensis* Miller, Propoli WSEP-70 and small quantities of *Ravensara* Niaouly essential oil were added to obtain a nasal spray with no preservatives and no alcohol, suitable even for kids. To evaluate the effectiveness of the product, the ENT specialist took, before starting the treatment cycle, a nasal cytology sample from the middle part of the inferior turbinate using nasal scraping. The sample was then placed on a glass slide and stained using the standard technique of May-Grünwald Giemsa. After preparation, the smear was analysed using a 1000x oil immersion optical microscope Nikon Eclipse 200 (Nikon Instruments S.p.a., Florence, Italy), according to Gelardi observation method [10].

The lemon pulp extract was prepared by means of the Extractor Naviglio LAB series model 500 cc (Atlas Filtri srl, Padua, Italy); demineralized water and 2 kg of lemons (12 lemons). The flavedo was removed from the lemons, and the fruits were subdivided into 4 groups (three lemons per group); then, the albedo was also removed, and each lemon was cut into eight pieces. The pieces from three lemons were placed into a food grade polyethylene bag, with large meshes. The bag was then put into the extraction chambers of the extractor, and, after the addition of 500 mL of demineralized water, it was tightly closed. In our experience has been used a total extraction cycle equal to 1 hour (30 cycles); static phase: 1 minute; dynamic phase: 5 piston strokes (1 min.).

Standardizing the extraction time is a crucial parameter to obtain reproducibility in the quantity of drug extracted, as has been demonstrated in works reported in the literature [11].

 The liquid extract recovered at the end of the extraction process has been pasteurized for 30 minutes at its boiling point; it was then transferred into a glass bottle and stored in refrigerator at 4°C until the time of its use so that the chemical and microbiological stability of the lemon extract is guaranteed for over six months.

100 patients were chosen, both male and female, with an average age of 34 years (min 3, max 79 years), suffering from vasomotor allergic rhinopathy and not undergoing treatment with corticosteroids or antihistamines (systemic or topical), or with nasal decongestants. Patients with structural defects (nasal septum deviation, alterations of the nasal valve dynamics, polyposis, and infectious rhinopathies), pregnant and lactating women, and patients with oncological and autoimmune diseases were excluded from the study. Patients were later divided into two homogeneous groups: group A was administered the spray based on lemon extract; group B was administered saline solution isotonic. All subjects completed the trial.

Special attention was paid to the sampling and cytological processing phases of the study. The cytological sampling was performed by means of the scraping technique, by smearing a rhinoprobe 2-3 times (nasal scraping) on the surface of the mucosa in the middle area of the inferior turbinate. For children under 5 years old, a sterile buffer soaked with sterile physiological solution was used and then smeared on the medium part of the inferior turbinate. The material sampled in this way was then transferred on a standardized microscope glass slide and spread accurately to obtain a thin layer. The glass slide was then stained using the May-Grünwald-Giemsa method, which we prefer because it can color all the cellular components commonly present in normal and immune/inflammatory conditions. Moreover, an overall clinical profile was defined for each subject, indicating a number from 0 (no symptoms) to 3 (all allergic symptoms developed), which represents the patient's score.

**Figure 1:**
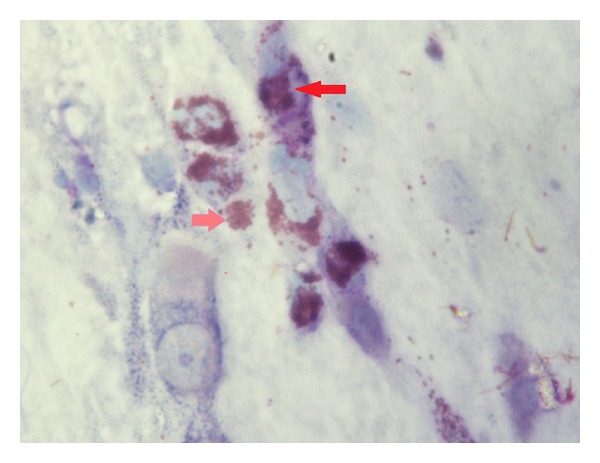
Rhinocytogram before the treatment in a patient with score 3. Eosinophils and mast cells can be noticed, and wide areas of mastocytary (red arrow) and eosinophil (pink arrow) degranulation.

Observing the sample under an optical microscope Nikon Eclipse 200 magnified by 1000 in oil, we proceeded with a reading by field, examining the entire surface of the slide to detect the cellular elements most relevant for our diagnostic purposes (neutrophils, eosinophils, lymphocytes, and mast cells). At the same time, the cells of the olfactory epithelium were observed. A basic cytology was performed first, at the time when the patients were included in the study (Figure 1). In the following 10 days, a treatment was administered with the nasal spray (two puffs equal to 0.14 mL, 3 times/day). In 0.14 mL, are present: 0.014 mL of lemon extract; 0.042 mL of pure *Aloe* juice; 0.0007 mL of *Ravensara* essential oil, 0.0007 g of Propoli WSEP-70, and 0.00042 mL of Niaouly essential oil.

**Figure 2:**
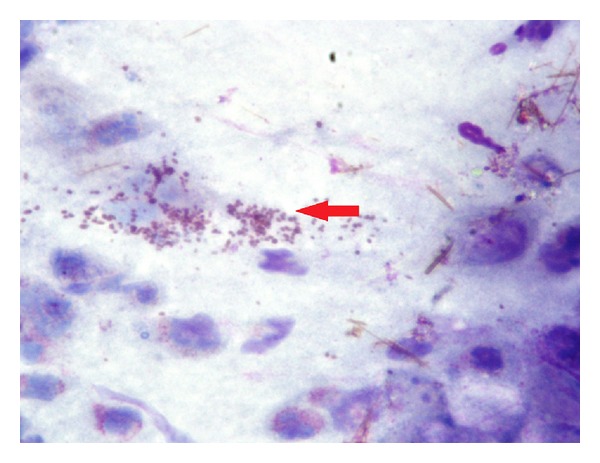
Rhinocytogram of the same patient with score 3 after 15 days of treatment (actual: score 0). Rare mast cells degranulation (red arrow).

**Figure 3:**
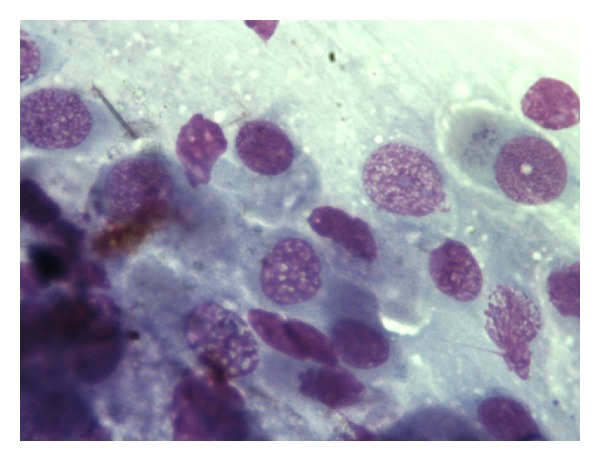
Rhinocytogram of the same patient with score 3 after 30 days (actual: score 0). The absence of inflammatory cells can be noticed.

 A check nasal cytology was made at the end (Figure 2). A rhinocytological control was subsequently made on all patients after 30 days of therapy (Figure 3).

 In the execution of the study a homogenous group of ten people aged between 5 and 65 years was recruited as a control. This group was administered with physiological solution (saline solution) of equal quantity to the study group.

**Figure 4:**
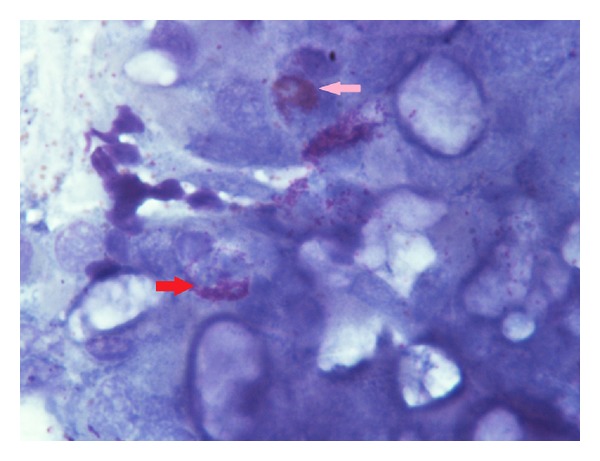
Control group (score 3) rhinocytogram before treatment. Eosinophil cells (pink arrow) and mast cells (red arrow) observed with large areas of degranulation.

**Figure 5:**
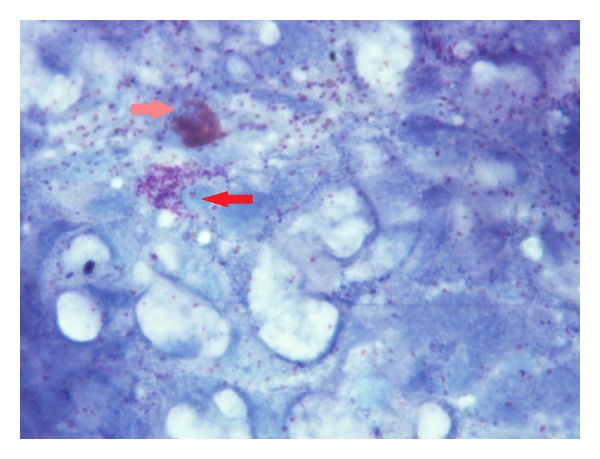
Control group (score 3) rhinocytogram performed at fifteen days of treatment. It is not an observed modification of eosinophil cells (pink arrow) and mast cells (red arrow).

**Figure 6:**
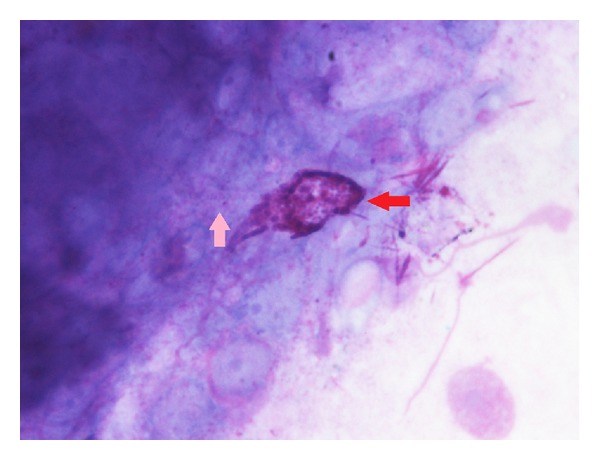
Control group (score 3). Rhinocytogram post-treatment. The cytologic pattern is almost unchanged from the initial assessment. Eosinophilic (pink arrow), mast cells (red arrow).

The control group was observed at the end of treatment, and, not only the persistence of inflammatory cells was unchanged, (Figures 4, 5, and 6) except for a slight reduction of neutrophils, due to the cleaning action of the water, but also the persistence of allergic symptoms of a general type in all subjects examined (totality of cases) was unchanged.

 For the statistical evaluation of experimental results, Student's *t*-test has been used.

## 3. Discussion

Different studies witness daily the efficacy of lemon extract [12–14] on the nasal mucociliary clearance and to the properties of water-soluble flavonoids on venous microcirculation. There are accounts in the literature that lemon juice is amongst the most powerful natural antiseptic and bactericide; it is beneficial in ear infections and in colds and has a certain efficacy in treating inflammations of the throat, mouth ulcers, gingivitis, and inflammations of the tongue.

*Aloe* juice has an antiallergenic and antiallergic effect, proving to be highly efficient in most cases; moreover, the effect of *Aloe* tincture has long been known in nasal-oropharyngeal infections [15, 16]. The essential oil of Niaouly represents an effective protective agent in the treatment of infections to the breathing tracts, because its vapors have bactericidal, immunostimulant, hyperemizing, mucolytic, and balsamic properties [17, 18]. 

Thanks to its great antibacterial, antiviral, and expectorant properties, the essential oil of *Ravensara* represents an excellent remedy in infection to the breathing tracts [19].

The propolis WSEP-70 standardized at 10% (w/w) in quercetin and 75% (w/w) in total polyphenols expressed in galangin is an extract of water-soluble propolis, with the capacity of favoring the natural defense of the organism against inflammatory disturbances to the nasal and oropharyngeal cavities [20, 21]. 

Upon the first observation, the rhinopathic subjects displayed the typical symptoms of the allergy: nasal obstruction, rhinorrhea, and sneezing, more or less accentuated. Hypertrophic turbinates of a bruised-pale coloring appear with the rhinoscopy. The patients subjected to the treatment, from a subjective-overall symptomatology point of view, displayed the following: 63 patients a score of 3, equal to 63% of the subjects examined; 21 patients a score of 2, equal to 21%; 11 a score of 1, equal to 11%, and 5 a score of 0, equal to 5% (Figure 8).

Upon the evaluation of the initial rhinocytogram, a rich neutrophilic component was documented on a whole, with a discreet but constant representation of eosinophils and mast cells (in nine cases, even rare lymphocytes). In all the subjects examined, in the first ten days, a marked regression of the symptoms was noticed, with a discreet return to norm of nasal objectivity and of individual symptoms; it proved necessary to increase the times of daily administration in only two cases, due to a persistent allergic symptomatology. When observed under the microscope, the absence of neutrophils and lymphocytes was noticed, with a reduction by more than 50% of the eosinophils and of the mast cells. In certain cases, rare degranulations of the eosinophils and mast cells were observed (Figure 3).

**Figure 7:**
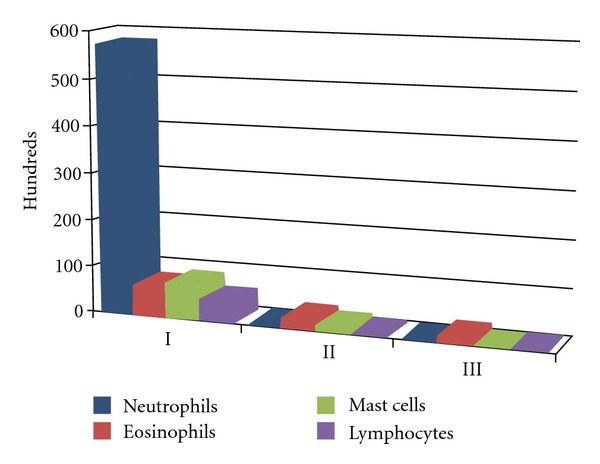
Total progress of inflammatory cells. (I) treatment start; (II) after 15 days; (III) after 30 days.

**Figure 8:**
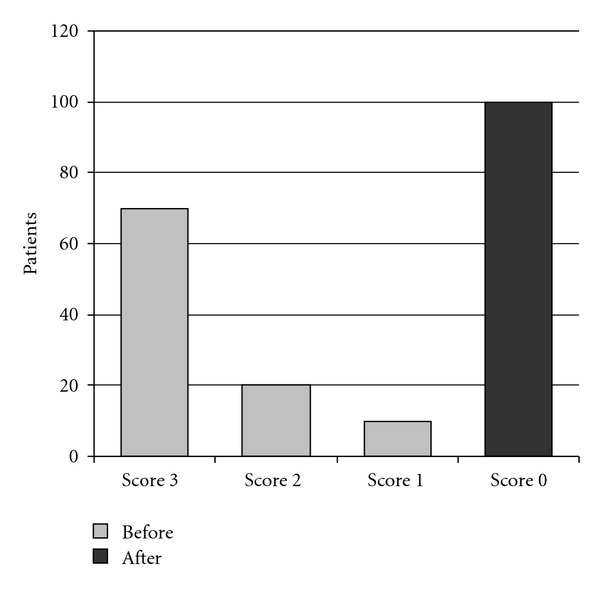
Overall subjective before-after treatment score for the totality of patients.

At the end of the therapy, all the subjects displayed an individual symptomatology equal to 0 (no symptoms). This was confirmed both by the objective local examination, which documented a considerable improvement of the mucosa, and by the rhinocytogram, which detected a shear total reduction of the inflammatory cells (Figure 7).

**Figure 9:**
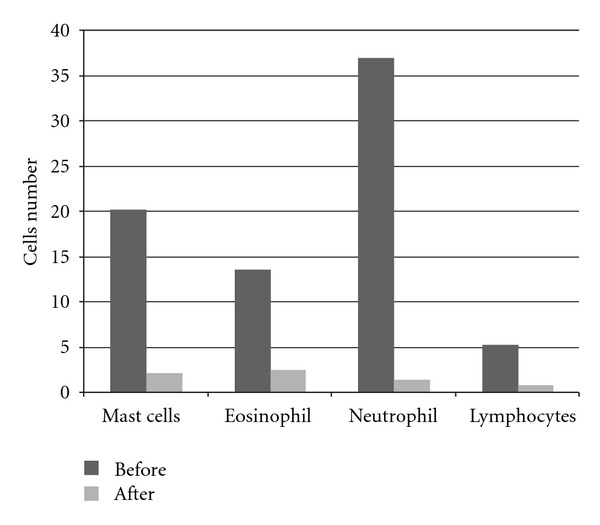
Inflammatory cells incidence before and after terapy (*P* < 0.01).

 In two cases, rare mast cells and eosinophils with relative degranulations could be observed (*P* < 0.01) (Figure 9). In the clinical case that displayed a worsening of the symptomatology, during the nasal cytology, a presence of several mycotic colonies was discovered, as well as the presence of an inflammation of crusty rhinitis during the objective examination; this symptomatology may be the consequence of not having undergone regular therapy.

 A possible placebo effect was tested, as reported in “clinical trials” section, by applying repeated and prolonged physiological solution that did not induce in the control group, as was to be expected, a significant change in clinical symptoms and in objective pictures of rhinocytograms.

The experimentation with the nasal spray pointed out, in addition to the reported results concerning the cytological exam, immediate effects, such as decongestion of a clogged nose and a consequentially improved breathing. From the first applications of the product, abundant elimination of liquids is displayed, which contributes toward off deposits of mucous and irritating substances, favoring an accurate nasal hygiene.

 The natural substances present in the spray have undoubtedly shown anti-inflammatory properties and are therefore recommended in all those pathologies, where the use of cortisone may be counter indicated. The results of this case study, which agree with the data described in the literature, demonstrate that the study of the nasal mucosa in the diagnostic approach on patients suffering from allergic rhinopathy is, by way of nasal cytology, an undoubtedly valid method, in addition to being a well-tolerated procedure that is simple to perform. The forms studied have documented a cytological picture represented by a relevant level of pathognomonic neutrophils, eosinophils, and mast cells of the allergic forms [22–26]. The lymphocytes present are probably tied to previously onset viral infections.

## 4. Conclusion

 Topical administration of the lemon-based spray has pointed to the shear total disappearance of the eosinophils granulocytes and mast cells, with the persistence of a few neutrophils or lymphocytes, and extremely rare eosinophil and metachromatic granules over the period of the treatment. The analysis of the rhinocytograms perfectly parallels, the improvement of the clinical picture. Though the number of cases studied is small to draw conclusive considerations, the positive results obtained allow us to assume that the spray surely has an anti-inflammatory effect. In conclusion, we feel that it is safe to affirm that the spray can be used with all those adult patients for whom traditional therapy is contraindicated, and with children, considering that it is free of alcohol-based substances. Furthermore, we can argue that the nasal cytology may be a useful method and a valuable tool to assess not only the clinical phases of an inflammatory disease of the nose, but also to monitor the effects of therapy on a component of the inflammatory cells, whose reduction is a guarantee of efficacy.
